# ^13^C‐Labelled Glucose Reveals Shifts in Fermentation Pathway During Cathodic Electro‐Fermentation with Mixed Microbial Culture

**DOI:** 10.1002/cssc.202401033

**Published:** 2024-11-11

**Authors:** Gaia Salvatori, Ottavia Giampaoli, Angela Marchetti, Alfredo Miccheli, Bernardino Virdis, Fabio Sciubba, Marianna Villano

**Affiliations:** ^1^ Department of Chemistry Sapienza University of Rome P.le Aldo Moro 5 00185 Rome Italy; ^2^ NMR-Based Metabolomics Laboratory Sapienza University of Rome P.le Aldo Moro 5 00185 Rome Italy; ^3^ Department of Environmental Biology Sapienza University of Rome P.le Aldo Moro 5 00185 Rome Italy; ^4^ Australian Centre for Water and Environmental Biotechnology The University of Queensland Brisbane QLD 4072 Australia

**Keywords:** Cathodic electro fermentation (CEF), Short chain carboxylic acids (SCCA), Mixed microbial cultures (MMC), Fully labelled glucose (U-^13^C), Nuclear magnetic resonance (NMR)

## Abstract

Cathodic Electro‐Fermentation (CEF) is an innovative approach to manage the spectrum of products deriving from anaerobic fermentation. Herein, mixed microbial culture fermentation using a ternary mixture containing labelled ^13^C glucose and non‐labelled acetate and ethanol was studied to identify the role of polarization on the metabolic pathways of glucose fermentation. CEF at an applied potential of −700 mV (*vs*. SHE, Standard Hydrogen Electrode) enhanced the production yield of acetate, propionate, and butyrate (0.90±0.10, 0.22±0.03, and 0.34±0.05 mol/mol; respectively) compared to control tests performed at open circuit potential (OCP) (0.54±0.09, 0.15±0.04, and 0.20±0.001 mol/mol, respectively). Results indicate that CEF affected the ^13^C labelled fermented product levels and their fractional ^13^C enrichments, allowing to establish metabolic pathway models. This work demonstrates that, under cathodic polarization, the abundance of both fully ^13^C labelled propionate and butyrate isotopomers increased compared to control tests. The effect of CEF is mainly due to intermediates initially produced from the glucose metabolic transformation in the presence of non‐labelled acetate and ethanol as external substrates. These findings represent a significant advancement in current knowledge of CEF, which offers a promising tool to control mixed cultures bioprocesses.

## Graphical abstract

Cathodic electro‐fermentation (CEF) with undefined mixed microbial cultures in the presence of fully labelled ^13^C glucose and non‐labelled ethanol and acetate as co‐substrates, affected the abundance of ^13^C labelled fermented products and their fractional ^13^C enrichments. Changes of the ORP (Oxidation‐Reduction Potential) was the main trigger of the process.

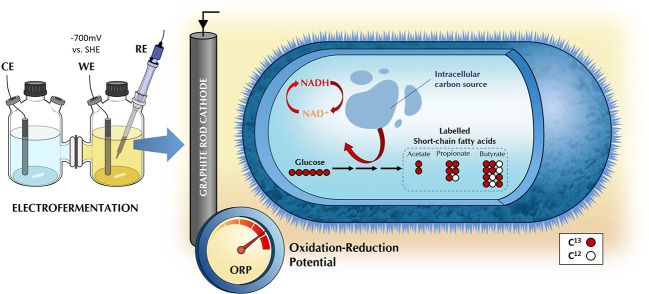

## Introduction

Bioproducts of anaerobic fermentation processes, especially short‐ and medium‐chain carboxylic acids (i. e., organic acids with a number of carbon atoms lower than six and between six and twelve, respectively) have attracted considerable interest for their wide range of applications, including as biopolymer building blocks (e. g., polyhydroxyalkanoates),^[1]^ carbon sources for biological nitrogen removal from wastewater,^[2]^ and as fuels in microbial fuel cells aimed at electrical power production.^[3]^

Acidogenic fermentation (AF) is the most common process to obtain high yields of conversion of organic wastes into short‐chain carboxylic acids (SCCA).^[4]^ However, currently, there are no mixed culture‐based AF processes applied at the commercial scale. Generally, the optimization of the AF process requires the achievement of specific goals such as maximizing acids yield and concentration.^[5]^ On the other hand, anaerobic digestion (AD) is a mature technology that uses organic waste as substrates (including complex waste such as animal manure, sewage sludge, crop residues, etc.) for biogas production.[^5^, ^6^] SCCA are produced during AD as fermentation intermediates, and their production has recently been receiving attention due to their higher commercial value and a wider range of applications than AD products such as biogas and compost.[^7^, ^8^] This has prompted increasing research interest into finding methods to manipulate AF and control the spectrum of products and their relative distribution.

In this respect, electro‐fermentation (EF) has been proposed as an appealing strategy to control microbial fermentative metabolism using a polarized electrode strategically placed within the reaction medium.[^9^, ^10^, ^11^] Depending on the conditions, the electrode can be operated to serve as either 1) an electron sink (anodic EF, also referred to as AEF); 2) an electron donor (cathodic EF, also referred to as CEF); or 3) to control the oxidation‐reduction potential (ORP) when the current is not the product of interest. Furthermore, in this last option, the polarized electrode is suggested to affect the redox state of cofactors such as NADP^+^ and NADPH, which alters the relative distribution of reduced and oxidized products.[^10^, ^12^] Some reports have shown how CEF process can enhance the yield of production of reduced compounds (e. g., SCCA). Specifically, it has been suggested that the cathode supplies electrons, which are then used by the microbial cells to increase the intracellular NADH, thereby inducing the production of more reduced metabolites.[^9^, ^13^]

Interestingly, the shift towards reduced products in the CEF systems has been identified in studies employing both pure and mixed microbial cultures. For example, García Mogollón *et al*. reported of an increased yield of production by a factor of 2.2 – with respect to the conventional fermentation control – of acetone, butanol, and ethanol by *Clostridium saccharoperbutylacetonicum* N1–4 when the electrode was poised at −600 mV (vs. Ag/AgCl).^[14]^ Similarly, Zhang and co‐workers have shown an enhancement in butyric acid production under CEF conditions (at −0.80 V vs. Ag/AgCl).^[13]^ In particular, the production and specific yield of butyric acid in the CEF system reached a value of 5.54 g/L and 0.41 g/g_RiceStraw,_ which were higher than those obtained in the open circuit (OC) system by 17.4 % and 28.1 %, respectively. Furthermore, under CEF conditions, the mixture of fermented products was enriched into butyric acid up to 52.8 %.

Similar results were also obtained in previous studies whereby the electrode (polarized at −700 mV *vs*. SHE) triggered a 20‐fold increase (relative to OCP, Open Circuit Potential conditions) in the yield of isobutyrate production (0.43±0.01 *vs*. 0.02±0.02 mol/mol glucose) during the anaerobic fermentation with Mixed Microbial Cultures (MMC).^[15]^

During the CEF process, the electrical current is not the product of interest. As such, it is seldom reported in most studies.^[10]^ The low cathodic current usually measured in these systems suggests that an unbalance of cofactors redox state (e. g. NADH or NADPH and ferrodoxin)[^16^, ^17^] is a major trigger in manipulating the distribution or increase in SCCA production.

Despite the large, yet sometimes contrasting, body of evidence indicating the viability of EF‐based approaches to alter the fermentative metabolism, the underlying mechanism ‐ particularly at the physiological level ‐ remains largely elusive.

In this work, we investigated SCCA production during a CEF process (at −700 mV *vs*. SHE) using anaerobic MMC fed with a ternary mixture of organic substrates. Changes in MMC fermentation products, monitored over a 16‐day period, highlighted that CEF induced an increase in the acetate, propionate and butyrate yields compared to the open circuit controls. Moreover, the use of fully labelled ^13^C glucose as the carbon source enabled tracking of the production of intermediate fermentation products using NMR spectroscopy, providing insight into the metabolic pathways involved. Specifically, by tracking the labelled fermented products (mostly acetate, propionate, butyrate), we were able to establish a correlation between the NADH‐dependent steps in the fermentative metabolic pathway and the applied electrochemical conditions.

## Results and Discussion

Cathodic electro‐fermentation (CEF) was studied with a ternary mixture of substrates, consisting of ethanol, acetate, and fully labelled ^13^C glucose, to identify the main changes in metabolic pathways that may be induced by the presence of a poised electrode during MMC fermentation. Results were compared with a similar fermentation system, where instead the electrode was kept at open circuit. Substrate composition and ratio used here are the same as in a previous study wherein glucose was not labelled^[15]^ and a significant increase in iso‐butyric acid production was observed during similar cathodic EF conditions. The substrates were added to the biotic compartment of H‐type cells (i. e., the compartment hosting the working electrode).

When considering only the ^12^C and ^13^C isotopes in the carbon backbone of a molecule *M* with *n* carbon atoms, an isotopomer of *M* is one of the *2* ^*n*^ possible labelling states in which this molecule can be encountered. However, when examining the labelling distribution in a biological system, the actual number of observed isotopomers is often a fraction of the total possible states. This is due to the biochemical pathways involved in the metabolism of the labelled substrate. Here, we used this feature to shed light into the fermentation pathways.

**Figure 1:**
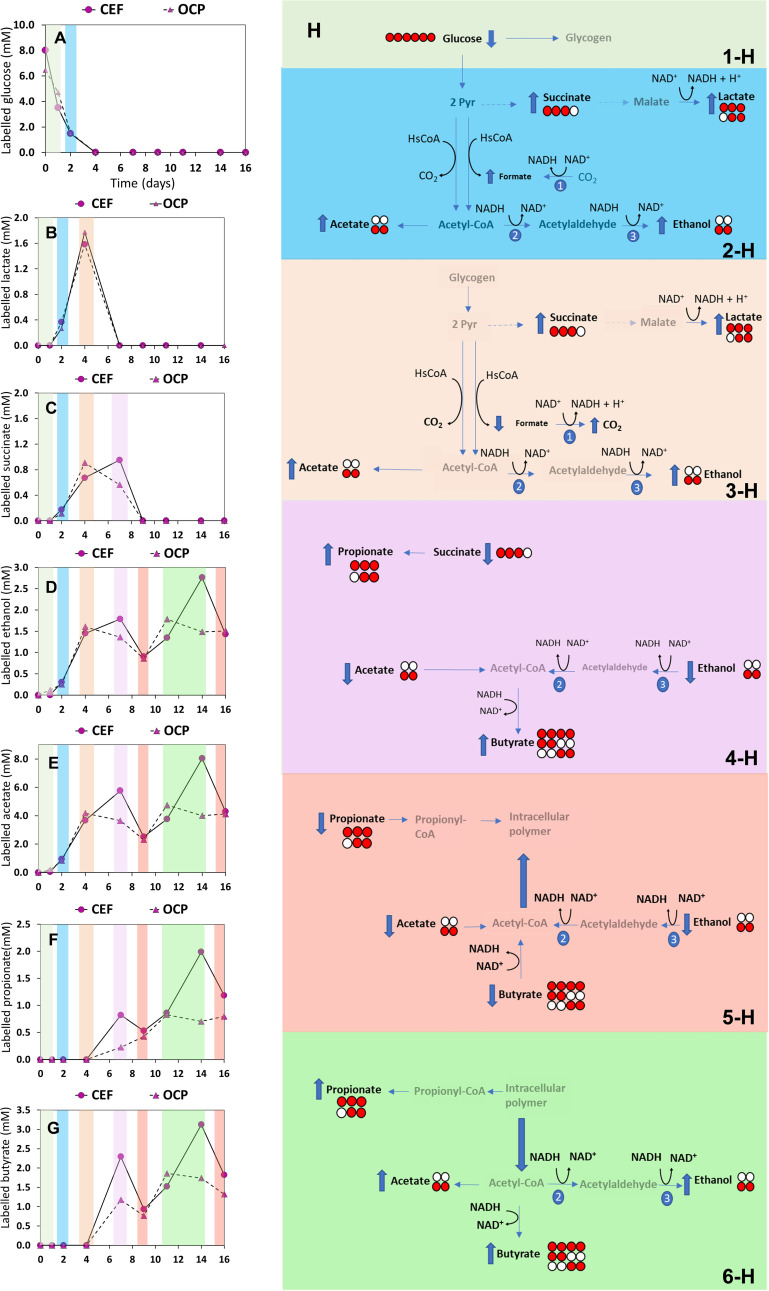
Time course of labelled **[U‐^13^C_6_]** glucose (A), ^**13**^ **C** labelled lactate (B), ^**13**^ **C** labelled succinate (C), ^**13**^ **C** labelled ethanol (D), ^**13**^ **C** labelled acetate (E), ^**13**^ **C** labelled propionate (F), and ^**13**^ **C** labelled butyrate (G) in the CEF and OCP tests; and the metabolic pathways of labelled glucose ^13^C fermentation during the operative days (H): metabolic pathway day 0 and 1 (1‐H); metabolic pathway day 2 (2‐H); metabolic pathway day 4 (3‐H); metabolic pathway day 7 (4‐H); metabolic pathway day 9 and 16 (5‐H); metabolic pathway day 11 until 14 (6‐H). (Numbers 1, 2, and 3 represent the formate dehydrogenase, acetaldehyde dehydrogenase, and ethanol dehydrogenase; respectively). The concentration of labelled compounds refers to the sum of all isotopomers which have been detected. For each isotopomer the red and white circles refer to the labelled (^13^C) and non‐labelled (^12^C) carbon, respectively.

Figure 1 reports the concentration profiles of labelled ^13^C‐glucose and fermentation products referred to one model test performed under OCP and CEF conditions. ^13^C‐Glucose was consumed at a similar rate under both conditions (Figure 1A). Lactate also showed the same trend in both conditions with a very similar maximum concentration at day 4 (Figure 1B) of 1.58 and 1.77 mM for EF and OCP tests, respectively. Instead, succinate reached its maximum concentration at day 4 for the OCP test (0.91 mM), whereas it reached its maximum a few days later (on day 7) for the CEF test (0.95 mM) (Figure 1C)

### Distribution of ^13^C‐Labelled Products During MMC Fermentation

The main results in terms of ^13^C labelled fermentation products obtained in a typical test under OCP conditions are reported in Figure 1. ^13^C NMR spectra for an OCP experiment (Figure S1) were characterized by a complex pattern of signals indicating the presence of multiple labelled isotopologue species in significant amounts (Table S1). Thus, the differential ^13^C‐isotopomer traces observed could be related to both qualitative and quantitative differences in the metabolic networks, leading to the synthesis of individual metabolites.

Each molecule of [1,2,3,4,5,6‐^13^C_6_] glucose, which was provided as substrate on day 0 (Figure 1: 1‐H), was metabolized, through the glycolytic pathway, to two molecules of [1,2,3‐^13^C_3_] pyruvate (which was not observed in the analyzed samples, being it an intracellular intermediate) with the production of ATP and NADH. Glucose concentration rapidly decreased, and it was below the detection limit in the medium after day 4.

In order to regenerate NAD^+^ and follow up with the production of ATP through the glycolytic pathway, [1,2,3‐^13^C_3_] pyruvate could be converted to [1,2‐^13^C_2_] acetaldehyde by pyruvate decarboxylase and further reduced to [1,2‐^13^C_2_] ethanol.^[18]^ Another possible pathway of [1,2,3‐^13^C_3_] pyruvate could also lead to [1,2‐^13^C_2_] acetyl‐CoA after oxidative decarboxylation by pyruvate dehydrogenase and then be released in the medium as [1,2‐^13^C_2_] acetate. Both [1,2‐^13^C_2_] ethanol and [1,2‐^13^C_2_] acetate were observed to increase between day 2 to 4 according to a similar trend (Figure 1D and E), followed by a decrease until day 9 before further increasing to a maximum at day 11 and then remaining almost unchanged (at around 1.61 mM and 4.18 mM for labelled ethanol and acetate, respectively) until day 16 (last sampling day).

[1,2,3‐^13^C_3_] succinate was detected in the reaction media between day 2 and day 7 (Figure 1: 2‐H, 3‐H, 4‐H) with a maximum (0.91 mM) measured at day 4 (Figure 1C). Succinate synthesis derived from the reductive branch of the 3‐HP cycle starting from [1,2,3‐^13^C_3_] pyruvate through the addition of non‐labelled CO_2_ (which was used to guarantee the anaerobic condition as N_2_/CO_2_, 70/30 % v/v) to form oxalacetate via pyruvate carboxylase then malate, fumarate, and finally succinate (globally consuming 2 NADH).[^19^, ^20^] Starting from day 2, two lactate isotopomers, specifically [1,2,3‐^13^C_3_] lactate and [2,3‐^13^C_2_] lactate (Figure 1B), were detected in the fermentation medium at a maximum concentration (*ca*. 1.78 mM) at day 4, followed by its consumption and exhaustion by day 7. Since the two isotopomers were detected in a similar amount, their synthesis could be linked to a four‐carbon symmetric molecule with a labelling in 3 consecutive carbons. The only molecule possessing these characteristics in the reaction media is [1,2,3‐^13^C_3_] succinate, which can be oxidized to malate and then converted into lactate.^[21]^ Starting from day 7 the presence of other SCCA isotopomers, namely propionate and butyrate was also observed (Figure 1F and G). Interestingly, both products were observed after full consumption of glucose. The NADH dehydrogenases as well as the intermediate compounds implicated in the synthesis of these fermentation products are represented in Figure 1. Regarding propionate, the isotopomers [2,3‐^13^C_2_], [1,2,3‐^13^C_3_], and propionate in ^13^C natural abundance were detected. Both the ^13^C isotopomers could derive from the decarboxylation of [1,2,3‐^13^C_3_] succinate through methyl malonyl‐CoA decarboxylase[^22^, ^23^] since the molecule is chemically symmetrical. However, [1,2,3‐^13^C_3_] propionate could also derive from the reduction of [1,2,3‐^13^C_3_] lactate through the acrylate pathway.^[24]^

The isotopomers of propionate increased from day 7 reaching a plateau (0.80 mM) at day 11, which persisted until the end of the sampling.

This information is also corroborated by the fractional distribution of ^13^C in the fermentation products, reported in Tabe 1.

**Table 1:** ^13^C fractional enrichment of SCCA in the OCP fermentation tests (as mean values of replicated experiments).

Day	Propionate (%, mol/mol*)		Butyrate (%, mol/mol*)		
	[1,2,3]	[2,3]	[1,2,3,4]	[3,4]	[1,2]
7	0.52±0.03	0.48±0.02	0.35±0.02	0.33±0.02	0.32±0.02
9	0.54±0.03	0.46±0.02	0.36±0.02	0.32±0.02	0.32±0.02
11	0.55±0.03	0.45±0.02	0.39±0.02	0.32±0.02	0.29±0.01
14	0.50±0.03	0.50±0.02	0.39±0.02	0.30±0.02	0.30±0.02
16	0.50±0.02	0.50±0.03	0.33±0.02	0.33±0.02	0.33±0.02

*All enrichment fractions of the data are determined in relation to the total amount of compounds (non‐labelled+^13^C Labelled).

From the reported data it is interesting to note that [1,2,3‐^13^C_3_] propionate levels were higher than [2,3‐^13^C_2_] propionate on day 7, day 9, and day 11. This suggests that up to day 11, the fully labelled propionate derived from two different metabolic pathways, whereas towards the end of the fermentation (day 11–16) it was obtained mostly from succinate decarboxylation. This is interesting because the conversion of lactate to propionate is a process requiring the consumption of NADPH,^[24]^ as such, it can possibly be influenced by the EF process (see below).

Regarding butyrate, three labelled isotopomers can be observed, namely [1,2‐^13^C_2_], [3,4‐^13^C_2_], and [1,2,3,4‐^13^C_4_] butyrate, as well as butyrate in ^13^C natural abundance. Butyric acid is synthesized through direct condensation (and successive reduction with global consumption of 2 NADH) of two acetyl‐CoA molecules.^[25]^ If both acetyl‐CoAs are labelled, the result is a fully labelled butyrate molecule, if only one is labelled, there is an equal probability to obtain [1,2‐^13^C_2_] or [3,4‐^13^C_2_] butyrate, while non‐labelled acetyl‐CoA would only yield non‐labelled butyrate. While [1,2‐^13^C_2_] acetyl‐CoA can derive only from the glycolysis of labelled glucose, non‐labelled acetyl‐CoA can derive from non‐labelled sources both intra (i. e., storage polymers or fatty acids) and extracellular (i. e., non‐labelled ethanol and acetate in the medium). In particular, both [1,2‐^13^C_2_] butyrate and [3,4‐^13^C_2_] butyrate could derive from the synthesis of one non‐labelled Acetyl‐CoA derived from the non‐labelled co‐substrate ethanol and one fully labelled [1,2‐^13^C_2_] acetyl‐CoA derived from [1,2‐^13^C_2_] acetate through the Reverse β Oxidation (RBO) chain elongation pathway.[^26^, ^27^]

Interestingly, all the butyrate isotopomers maintained the same relative ratio (Table 1) suggesting a similar contribution to the different butyrate synthesis pathways throughout the tests. More in detail, taking into account the sum of all butyrate isotopomers, they reached a first maximum on day 7, decreased on day 9, and then reached a second maximum on day 11, before slowly decreasing by the end of the test (Figure 1G).

This variability observed for the labelled fermentation products could be the result of the need, by the MMC, to regenerate NAD^+^ following the synthesis of pyruvate (through glycolysis), as well as acetate and succinate (through oxidative TCA pathway) due to cell respiration. Then, when the fermentation products achieved the maximum concentrations on days 7 and 14 (Figure 1F and G) there was also likely a maximum conversion of NAD^+^ to NADH. This combination, that is accumulation of SCCA and NADH, was previously reported to trigger the production of an intracellular polymer.[^28^, ^29^] Generally, intracellular polymers are stored as energy and carbon reserves by the metabolic activity under different stress conditions. Our hypothesis here is that intracellular storage can also serve as a potential sink of reducing equivalents, thereby enabling the regeneration of NAD^+^ from NADH. After the first maximum conversion into SCCA (at day 7), a decrease in the same fermented products was observed. In order to explain the detected oscillatory trend and simultaneous consumption of SCCA (i. e., acetate, propionate and butyrate) and redox co‐factors (i. e., NADH), a storage polymer (e. g., polyhydroxyalkanoates or glycogen) as a parallel pathway was introduced, as demonstrated possibly occurring during anaerobic fermentation processes.^[30]^ Indeed, it has been reported previously of the possible glucose conversion into storage polymers, such as glycogen, by anaerobic mixed microbial cultures.^[31]^ Also, the conversion of transiently produced lactate and storage polymers into a mixture of SCCA has been hypothesized during the anaerobic degradation of glucose.^[32]^ Furthermore, the polymer consumption ‐ which requires ATP – likely released the NADH which could further convert to regenerate NAD^+^ and the intermedia of fermentation reaction (e. g., Acetyl‐CoA). At day 14, a significant difference was observed in terms of labelled SCCA between the OCP and CEF tests (Figure 1D–G). These results stressed that the OCP tests were characterized by a different redistribution in terms of reducing power consumption (i. e., NADH and storage polymers).

### Distribution of ^13^C Isotopomers Starting from [U‐^13^C_6_] Glucose During MMC Under Cathodic Electro‐Fermentation (CEF) Conditions

The tracking of labelled fermentation products (directly derived from fully ^13^C labelled substrates) turns out to be particularly interesting since their biosynthesis requires NADH‐dependent steps, which are typically influenced by the CEF process.^[11]^

In addition, the cathodic polarization is linked to a continuous regeneration of the reducing power (NADH), which could represent an important factor in increasing the SCCA production.^[13]^ Based on these considerations, the main idea of this study was to evaluate the effectiveness of the conversion of fully labelled glucose into SCCA as well as the distribution of the acids due to the presence of a polarized cathode. As previously discussed, the fully ^13^C labelled glucose provided as substrate was depleted after 4 days of operation in both OCP and CEF tests, but with a different response in terms of SCCA production. The first maximum concentration of fermentation products, achieved after 7 days, highlighted the key role of cathodic polarization on the possible alteration of the ORP in the fermentation broth, which possibly affected the NAD^+^/NADH cofactor balance (required for SCCA synthesis). In addition, a significant difference in terms of the fermented products was also obtained at day 14.

More in detail, with reference to a typical CEF experiment, acetate concentrations of 5.76 mM and 8.03 mM were detected during the first and second maximum (at day 7 and 14, respectively), and the concentration achieved during the second maximum was about twice the concentration determined in OCP test (4.01 mM). The same trend was observed in propionate and butyrate concentrations. The propionate concentrations under applied CEF conditions were 0.82 mM and 1.99 mM ‐ during the first and second maximum ‐ with respect to the OCP concentrations of 0.23 mM and 0.70 mM, respectively. Interesting results were also determined for butyrate production under CEF conditions. Figure 1G pointed out a great increase in the butyrate production for both maximums (2.29 mM and 3.12 mM, at day 7 and 14, respectively) with respect to the OCP test. These results are also confirmed by the previous study conducted with the same ternary mixture,^[15]^ but without the presence of the labelled compound (i. e., glucose).

Furthermore, the ^13^C fractional enrichment of SCCA in CEF tests (Table 2) highlighted a significant difference in terms of fully labelled propionate and butyrate with respect to the non‐fully labelled species. While in OCP samples fully labelled [1,2,3‐^13^C_3_] propionate was higher than [2,3‐^13^C_3_] propionate only on days 7, 9, and 11 (Table 1), in CEF experiments the [1,2,3‐^13^C_3_] propionate was always the predominant isotopomer, meaning that CEF stimulated the activity of the acrylate pathway, which is a reducing pathway as previously discussed. In addition, the butyrate isotopomers (Table 2) showed an increase in the productivity of fully labelled butyrate compared with [1,2‐^13^C_2_] or [3,4‐^13^C_2_] butyrate. These findings stressed the evidence that the polarized electrode affects all metabolic steps for the butyrate synthesis driven by the NADH cofactor.

**Table 2:** ^13^C fractional enrichment of SCCA in CEF samples (as mean values of replicated experiments).

Day	Propionate (%, mol/mol*)		Butyrate (%, mol/mol*)		
	[1,2,3]	[2,3]	[1,2,3,4]	[3,4]	[1,2]
7	0.59±0.03	0.41±0.02	0.40±0.02	0.31±0.02	0.28±0.01
9	0.53±0.03	0.47±0.02	0.39±0.02	0.32±0.02	0.29±0.01
11	0.53±0.03	0.47±0.02	0.41±0.02	0.32±0.02	0.27±0.01
14	0.61±0.03	0.39±0.02	0.40±0.02	0.33±0.02	0.27±0.01
16	0.52±0.03	0.48±0.02	0.46±0.02	0.28±0.01	0.26±0.01

*All enrichment fractions of the data are determined in relation to the total amount of compounds (non‐labelled+^13^C labelled).

In particular, to support the hypothesis that the CEF approach could have a modulating effect on the NAD^+^/NADH balance resulting in a shift of fermentation products,[^11^, ^33^] the main electrochemical parameters (i. e., current and electrode polarization) were monitored, while cyclic voltammetry (CV) measurements were performed at the end of the experiment to identify the possible presence of redox‐active components in the fermentation medium.

**Figure 2:**
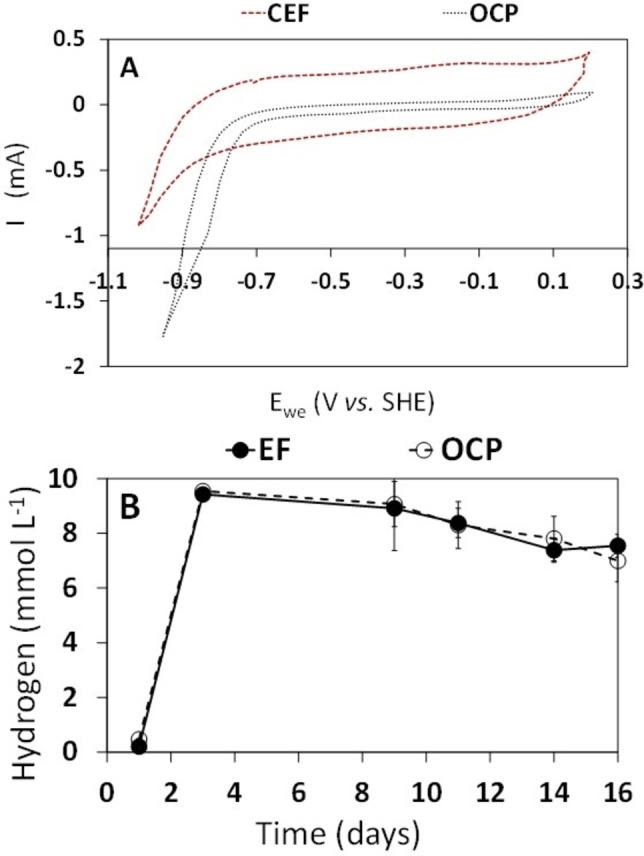
Cyclic Voltammetry (CV) trace (scan rate: 5 mV/s) recorded at the end of both CEF and OCP experiments (A). Time course of hydrogen concentration in both CEF and OCP experiments (as mean values of replicated experiments) (B).

The average current recorded during the CEF experiments accounted for −60±0.01 μA (Figure S2), resulting in an average current density of −5.0 μA/cm^2^. From an electron balance standpoint, this relatively low value of current density is not sufficient to explain the observed changes in the distribution of MMC fermentation products.[^9^, ^10^] CV traces (Figure 2A) recorded at the end of the experiments on the working electrode in the CEF experiments (red line) lack significant features (e. g., characteristic oxidation‐reduction peaks), and suggest the absence of redox‐active couples, for example resulting from the direct microbe‐electrode interaction^[34]^ or mediated by the presence of endogenous redox mediators in solution.^[13]^

Importantly, comparison between CV traces recorded on the CEF and OCP test showed no significant differences between the two systems, except for a clear catalytic wave observed in the OCP system at potentials below −750 mV, and likely ascribed to abiotic hydrogen generation, thermodynamically feasible in this potential range. This also suggests that during the electro fermentation, when a potential of −700 mV (*vs*. SHE) was applied, abiotic electrochemical H_2_ production was unlikely. In this view, the alteration of the ORP environment could make clear the effect of the polarized electrode on the SCCA enhancement. However, the measured hydrogen was negligible during the experiments and its concentration was very similar in CEF and OCP conditions, as reported in Figure 2B. Also, there was no difference in the pH values of all experiments, which as expected was approximately 5.5 (Figure S3). The main difference between CEF and OCP tests was in the potential of the working electrode, which was set at −700 mV in CEF conditions, whereas in the OCP test, it decreased over time (from day 0 to day 8) from +0.05 mV to −0.27 mV, a value at which it stabilized until the end of the experiments (Figure S3B).

It is possible that this difference in the electrode potentials affected the cofactor balance (NADH/NAD^+^), which is involved in the fermentation process as well as in the utilization of intracellular polymers possibly stored in both OCP and EF tests (Figure 1: 5‐H and 6‐H), as previously discussed. Indeed, the reduced power released during the intracellular polymer consumption could be used for NAD^+^ regeneration. Under cathodic polarization conditions, the specific electron transport paths for ATP synthesis could be more activated than in the OCP tests due to the reducing power supplied by the polarized electrode. Furthermore, the consumption of an intracellular polymeric carbon source ‐ which requires energy ‐ could be faster. Overall, CEF is considered a tool to manage the metabolic NADH steps and, in turn, a significant difference of the SCCA between EF and OCP ‐ during the second maximum on 14^th^ days (in correspondence with the consumption of a possible stored intracellular polymer) ‐ was achieved.

### Main Effects of CEF on the SCCA Production and Distribution

**Figure 3:**
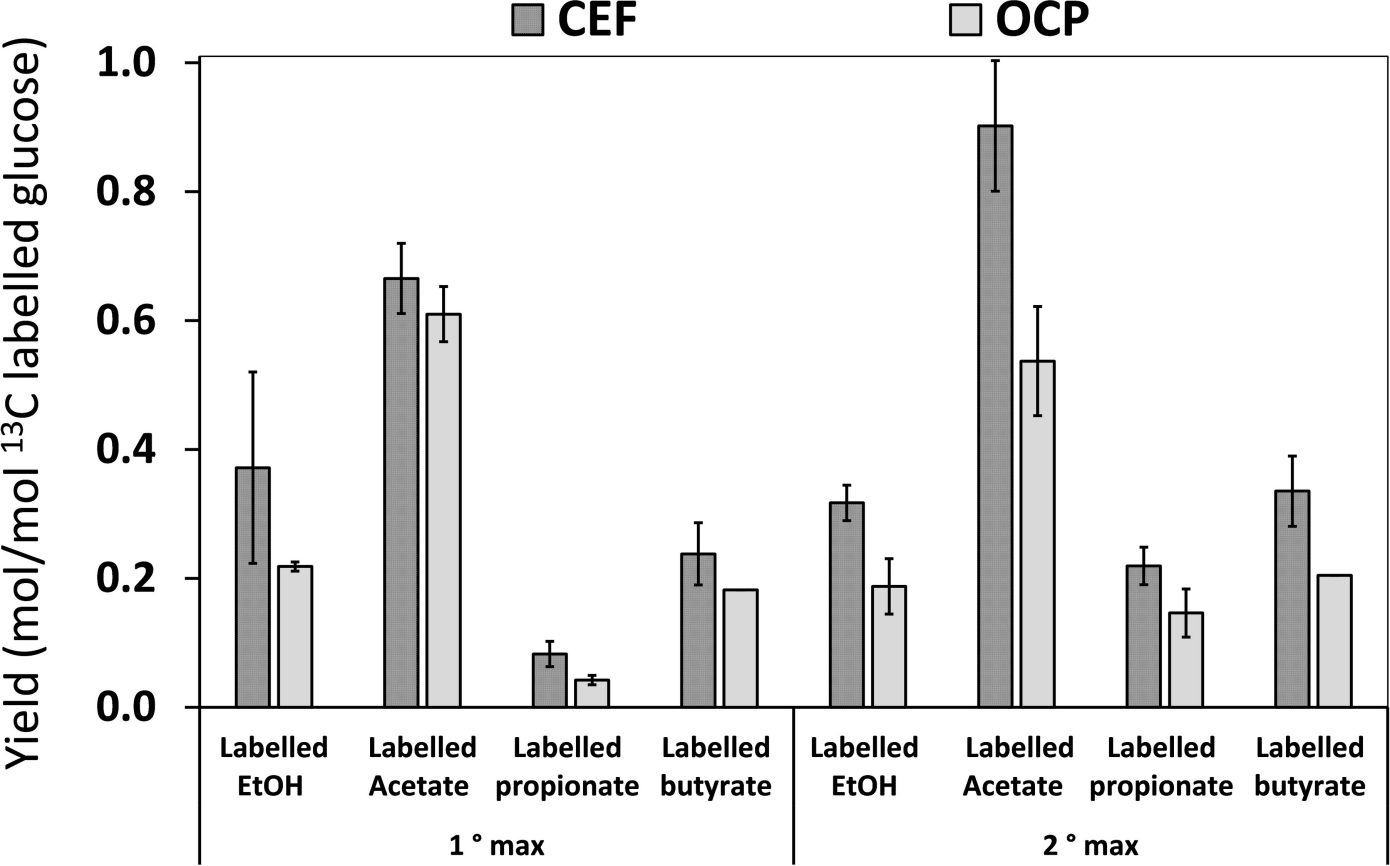
Yield of products formed from fully ^13^C labelled glucose fermentation under the polarized conditions (CEF) and in the absence of polarization (OCP). Error bars represent the standard deviation of replicated batch experiments.

As highlighted in the previous sections, the most evident difference in the CEF tests occurred after all the fully labelled glucose was depleted from the reaction medium, when the sum of ^13^C labelled fermentation products was significantly higher than in the OCP fermentation, possibly due to the major availability – at that time point ‐ of a full pool of intracellular reducing equivalents (e. g., as NADH). It is known that cathodic polarization stimulates reducing pathways.^[13]^ This is in good agreement with data obtained in this study, which indicate an increase of reduced species (e. g., propionate and butyrate) in CEF experiments with respect to the OCP controls (Figure 1). In addition, the maximum concentration achieved during the experiments (on day 7 and day 14) resulted in different yields of fermentation products between the CEF and OCP tests. Figure 3 shows the product yield calculated as the sum of all isotopomers of each labelled species with respect to the consumed labelled glucose (data are referred to mean values of replicated experiments). The consistently higher yields observed for the CEF tests compared to OCP tests corroborate the positive effect of the applied potential in promoting glucose conversion into fermentation products. Cathodic polarization likely allowed shifting in the NAD^+^/NADH ratio^[35]^ – under the polarization at −700 mV ‐ inducing higher production of fermentation compounds than in OCP controls. Furthermore, the CEF process could have prompted through the consumption of the intracellular carbon source (e. g., a stored polymer) – as shown in Figure 1: 5‐H and 6‐H – an increase of the NAD^+^, which represents a source to enhance the SCCA production. In this view, the production in CEF systems could be driven by a major conversion of NAD^+^ that implies an increase in the fermented products at the expense of OCP controls. More in detail, after the consumption of the intracellular carbon source (second maximum) the acetate, propionate and butyrate yields were 0.90±0.10, 0.22±0.03 and 0.34±0.05 (mol/mol) for CEF, respectively; and 0.54±0.09, 0.15±0.04 and 0.20±0.001 (mol/mol) for OCP, respectively.

Carbon and electron balances computed for both the CEF and OCP experiments, with respect to the labelled substrates and products, show that electro‐fermentation conditions resulted in a larger fraction of glucose carbon being directed towards fermented products (74±3 %, mol‐C_Products_/mol‐^13^C_GLU_) compared to the open circuit condition (54±4 %, mol‐C_Products_/mol‐^13^C_GLU_) (Figure S4A). Moreover, electron balance analysis indicates that despite the difference in carbon distribution induced by the CEF condition, the contribution of the cathodic current to the overall shift in the products profile during electro‐fermentation could only account for about 2 % of electron distribution (Figure S4B). This supports the hypothesis that the mechanism leading to a metabolic shift under electro‐fermentation conditions is likely due to changes in the redox environment rather than direct electron utilization to sustain fermentation.

Besides the changes occurring in the absolute amount of SCCA, another interesting change occurred in their isotopomeric distribution. As previously described, CEF stimulated the acrylate pathway (Table 2) ‐ activated during the fully labelled propionate synthesis – more than the control tests. Furthermore, another interesting finding ‐regarding the alteration of NADH cofactor during the synthesis of the SCCA‐ is related to the labelled butyrate isotopomer production. In particular, Table 3 points out the fractional enrichment of the fully ^13^C labelled butyrate for both OCP and CEF experiments. In CEF experiments the [1,2,3,4 ‐^13^C_4_] butyrate was the predominant isotopomer if compared with the control tests and the higher production of butyrate induced by the presence of the polarized electrode is fully in agreement with previous evidences obtained under very similar experimental conditions.^[15]^

**Table 3:** ^13^C fractional enrichment of SCCA in CEF and OCP samples referred to fully labelled butyrate (as mean values of replicated experiments).

Day	[1,2,3,4] Butyrate (% mol/mol*)	
	OCP**	CEF**
7	0.35±0.02	0.40±0.02
9	0.36±0.02	0.39±0.02
11	0.39±0.02	0.41±0.02
14	0.39±0.02	0.40±0.02
16	0.33±0.02	0.46±0.02

* All enrichment fractions of the data are determined in relation to the total amount of compounds (non‐labelled+^13^C labelled). ** Statistically significant differences between fully labelled butyrate detected in OCP and EF tests, according to Student′s t‐test (p<0.05).

These results suggest that the cathodic polarization stimulates the mechanism that implies the condensation of two labelled acetyl‐CoA and, in turn, the metabolic steps that are NADH dependent. Importantly, the metabolic pathway for the synthesis of [3,4‐^13^C_2_] butyrate and [1,2‐^13^C_2_] butyrate triggered by RBO does not seem to be favoured under CEF.

## Conclusions

This study analyzed the CEF (Cathodic Electro‐Fermentation) process by tracking the metabolism of fully labelled glucose in the presence of co‐substrates ethanol and acetate, to identify the key metabolic pathways through tracking the ^13^C isotopomers produced during the mixed culture fermentation process. CEF allowed to steer the fermentation pathways in which the NADH cofactor is involved resulting in a greater yield of the fermented products (i. e., ethanol, acetate, propionate, and butyrate), despite acetate and ethanol being present as substrates. Both CEF and OCP experiments showed an oscillatory trend in terms of SCCA production over the time (until 16 days) after complete ^13^C glucose depletion (not steady‐state isotopic condition). Here, for both conditions, a stored polymer as an energy reserve and sink of the reducing power was introduced in the model of metabolic fermentative pathway with MMC. This suggests that after the depletion of the main labelled carbon source (i. e., ^13^C glucose), the fermentative process can proceed using the intracellular polymer to promote SCCA production. Furthermore, the CEF effect is still evident (during the second maximum at day 14) stressing the result that the SCCA enhancement is likely due to a major conversion of NAD^+^ into NADH by the polarized electrode. Furthermore, a different isotopomeric abundance of the fully labelled propionate and butyrate was detected between OCP and CEF tests. These results underline how the polarized electrode (@ −700 mV vs. SHE) changes the ORP, and, in turn, the metabolic pathway involved during the fermented products synthesis. Overall, even though this study provides a significant advancement in current knowledge of the CEF process, further research with other labelled substrates is required to provide a deeper understanding of the pathways involved.

## Experimental Section

### Inocula

An anaerobic sludge deriving from a mesophilic pilot‐scale anaerobic digester treating the organic fraction of municipal solid waste was used as inoculum in all batch tests. Once collected, the anaerobic sludge was settled to remove the residual organic substrates contained in the supernatant, which was then replaced with mineral medium containing phosphate buffer at pH 5.5 to avoid the growth of methanogens (which would consume the fermentation products to generate methane), as described elsewhere.^[15]^

Prior to being used, the mixed microbial culture (MMC) was kept in borosilicate glass bottles (with a liquid volume of 1 L) under anaerobic conditions which were guaranteed by flushing the liquid and gaseous phase with a N_2_/CO_2_ (70/30, % v/v) gas mixture.

MMC was characterized in terms of biomass concentration, measuring the volatile suspended solid (VSS) which accounted for about 1.3±0.2 g/L.

### Reactor Configuration

**Figure 4:**
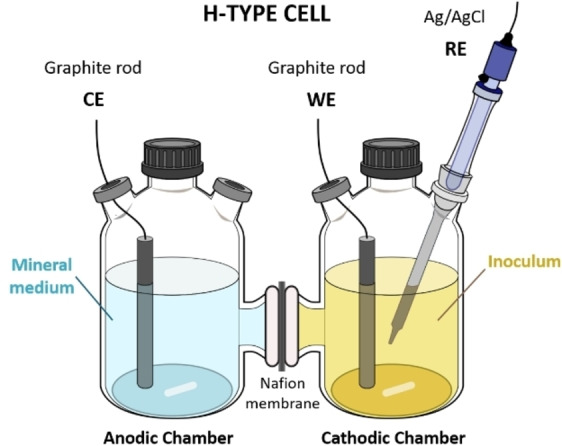
Scheme of the H‐type cells used throughout the experimentation.

Two‐chamber (H‐type) electrochemical cells were used to perform all the tests described in this study. Each cell consisted of two gastight borosilicate glass bottles (each with a total volume of about 270 mL), somewhat modified to include a glass bridge connecting the two bottles and hosting an ion exchange membrane (Nafion® 117 proton exchange membrane, PEM) with a 3 cm^2^ cross‐sectional area, and used to separate the electrolytes contained in the two compartments. Graphite rods (10 cm length, 5 mm diameter, Sigma‐Aldrich, Italy) equipped with titanium wires (0.81 mm diameter, Sigma‐Aldrich, Milan, Italy) were used as working and counter electrodes in the cathode and anode chamber, respectively. A KCl‐saturated Ag/AgCl reference electrode (+199 mV vs. the Standard Hydrogen Electrode, SHE) was placed in the cathode compartment to enable the application of a set potential to the cathode electrode. The PEM was previously treated by means of boiling in H_2_O_2_ (3 %, v/v) for 2 h, followed by boiling in deionized water for 2 h, successively in H_2_SO_4_ (0.5 M), and finally again in deionized water for 2 h. Prior to being used, the electrodes were treated with HCl (1 M) for 24 h and NaOH (1 M) for 24 h, each treatment followed by washing steps with distilled water. A schematic overview of the electrochemical cell configuration is reported in Figure 4.

### Electro Fermentation Batch Experiments

Mixed culture fermentation has been herein studied both in the presence (CEF tests) and in the absence (OCP tests) of electrode polarization. To investigate the effect of the electrode polarization on the distribution of products deriving from glucose fermentation, all experiments were performed in the same operating conditions except for the polarization of the cathode (serving as a working electrode), which was set at −700 mV vs. SHE (Standard Hydrogen Electrode) by means of a potentiostat (Biologic SP‐300, France) in CEF tests and was not controlled in OCP (Open Circuit Potential) tests. The value of the imposed cathode potential was chosen based on the results of a previous study.[^15^, ^33^] For OCP tests, performed as control experiments in the absence of electrode polarization, a digital multimeter (Keithley Instruments, Cleveland, OH) was used to periodically measure the value of the open circuit potential of the working electrode. Throughout the manuscript, all voltages are reported with respect to the SHE.

The cathodic chamber of each cell was inoculated with anaerobic sludge to an initial biomass concentration of 0.2 gVSS/L.

The carbon source fed to microorganisms in the cathodic chamber consisted of acetate (0.5 gCOD/L, with COD representing the Chemical Oxygen Demand), ethanol (0.5 gCOD/L), and fully labelled glucose ^13^C (1 gCOD/L), whereas the abiotic anode compartment was filled with mineral medium, consisting of (g/L): NH_4_Cl (0.5); MgCl_2_ ⋅ 6H_2_O (0.1); CaCl_2_ ⋅ 2H_2_O (0.05); K_2_HPO_4_ (8.7); KH_2_PO_4_ (61.22); 10 mL/L of a trace metals solution, and 1 mL/L of vitamins solution.^[15]^ The pH of the medium was buffered at 5.5 to inhibit the growth of methanogenic microorganisms (Figure S3A).

The liquid phase of each compartment (about 180 mL) was magnetically stirred at a rate of 150 rpm. Prior to the start of each experiment, anaerobic conditions were guaranteed by flushing both the liquid and the gaseous phases of each compartment with a N_2_/CO_2_ (70/30, % v/v) gas mixture.

Throughout the study, the two parallel CEF and OCP experiments were simultaneously started, and each condition was tested in duplicate.

### Analytical Methods

The concentration of microorganisms reported as VSS was determined according to the standard method APHA.^[36]^ Daily measurements of filtered liquid (0.22 μm porosity) samples were carried out for the determination of substrate consumption and the production of fermentation compounds by means of nuclear magnetic resonance (NMR) analysis.

Gas‐phase of each cell was sampled with a gas‐tight Hamilton syringe for hydrogen determination by injecting 50 μL of the gaseous sample into a Dani Master (Milan, Italy) gas chromatograph (stainless‐steel column packed with molecular sieve; N_2_ carrier gas; oven temperature at 100 °C; thermal conductivity detector, TCD, temperature at 150 °C). Headspace concentrations were converted to aqueous‐phase concentrations using tabulated Henry′s law constants.

### Sample Preparation for NMR Analysis

350 microliters of each sample were added to 350 microliters of trimethylsilyl propionic‐2,2,3,3‐d4 acid (TSP) ‐ D_2_O 2 mM solution (final concentration of 1 mM). This mixture of each sample was then vortexed and transferred into precision tubes.

### NMR Spectroscopy

#### 1D Experiments

1H‐NMR spectra were acquired at 298 K using a JEOL JNM‐ECZR spectrometer (JEOL Ltd, Tokyo, Japan) equipped with a magnet operating at 14.09 Tesla (600.17 MHz for 1H frequency and 150.91 MHz for ^13^C frequency) and a cryoprobe to improve the sensitivity. 1D 1H spectra were recorded with 64 k points and 128 scans, setting spectral width to 9.03 kHz (15 ppm), with an irradiation attenuator of 48 dB, a pre‐saturation pulse length of 2.00 s, relaxation delay of 5.72 s, for an acquisition time of 5.81 s.

1D ^13^C spectra were acquired at 298 K, employing an inversed gated pulse sequence. Relaxation delay was set to 7 s for all the spectra, acquiring a 32 k data point in 0.69 s. The spectral width was set to 47.4 kHz (250 ppm) and 3000 scans were collected for each spectrum to achieve an acceptable signal‐to‐noise ratio. An inverse‐gated decoupling pulse sequence was adopted since the observation of the ^13^C NMR signal′s fine structure relative to the coupling pattern between adjacent ^13^C, fundamental for isotopomer identification, crucially depends on the efficient removal of the large 1JCH scalar coupling interactions. This avoids the effects of NOE that could compromise ^13^C quantitative analysis. The decoupler was gated only during the acquisition of the ^13^C FID, after a single 90° detection pulse.

#### 2D Experiments

The identification step was achieved by two‐dimensional experiments 1H‐1H Homonuclear Total Correlation Spectroscopy (TOCSY), 1H‐13 C Heteronuclear Single Quantum Correlation (HSQC) on selected samples and confirmed by literature comparison. TOCSY experiments were recorded at 298 K with a spectral width of 15 ppm in both dimensions, using an 8 k × 256 data points matrix, repetition time of 3.00 s and 80 scans, with a mixing time of 80.00 ms. HSQC experiments were acquired with a spectral width of 9.03 KHz (15 ppm) in the proton dimension and 30 KHz (200 ppm) in the carbon dimension, using 8 k × 256 data points matrix for the proton and the carbon dimensions, respectively, with a repetition delay of 2 s and 96 scans.

### Data Analysis

^13^C and ^1^H processing and quantitation steps for one‐dimensional NMR spectra were achieved by using the ACD Lab 1D‐NMR Manager ver. 12.0 software (Advanced Chemistry Development, Inc., Toronto, ON, Canada); 2D‐NMR spectra were processed by using JEOL Delta v5.3.1 software (JEOL Ltd, Tokyo, Japan). All the NMR spectra were manually phased, baseline corrected and referenced to the chemical shift of the TSP methyl resonance at δ=0.00. The quantification of metabolites was obtained by comparing the integrals of their diagnostic resonances with the internal standard TSP integral and normalized for their number of protons, and then multiplied for two, in order to consider the dilution factor. The final concentration was expressed as mM.

All descriptive statistic values were calculated for each test using Microsoft Excel and error bars stand for the standard deviation of the mean of replicated tests. The statistical analysis was performed by analyzing data with the t‐student test.

### Fractional ^13^C Enrichment

In order to assess the changes occurring in the mixed microbial culture metabolism due to the application of CEF, it was necessary to quantify the ^13^C incorporated in specific carbons. This is done by expressing ^13^C incorporation as a fractional ^13^C enrichment by dividing the concentration of a specific isotopomer by the sum of the concentrations of all isotopomers, both ^13^C labelled and non‐labelled. The results were expressed as % of mol/mol. These enrichments were reported in Tables 1, 2 and 3.

## Conflict of Interests

The authors declare no conflict of interest.

1

## Supporting information

As a service to our authors and readers, this journal provides supporting information supplied by the authors. Such materials are peer reviewed and may be re‐organized for online delivery, but are not copy‐edited or typeset. Technical support issues arising from supporting information (other than missing files) should be addressed to the authors.

Supporting Information

## Data Availability

The data that support the findings of this study are available from the corresponding author upon reasonable request.
