# Supplementary material for: 13C‐Labelled Glucose Reveals Shifts in Fermentation Pathway During Cathodic Electro‐Fermentation with Mixed Microbial Culture
Source: ChemSusChem. 2024 Nov 11;18(2):e202401033. doi: 10.1002/cssc.202401033 (PMC11739826; doi:10.1002/cssc.202401033)
Supplement: Supplementary file 1 — Supporting Information [file CSSC-18-e202401033-s001.pdf]

ChemSusChem

Supporting Information

^{13}C-Labelled Glucose Reveals Shifts in Fermentation Pathway During Cathodic Electro-Fermentation with Mixed Microbial Culture

Gaia Salvatori, Ottavia Giampaoli, Angela Marchetti, Alfredo Miccheli, Bernardino Virdis, Fabio Sciubba,* and Marianna Villano*

SUPPORTING INFORMATION

Figures S1-S4
Tables S1

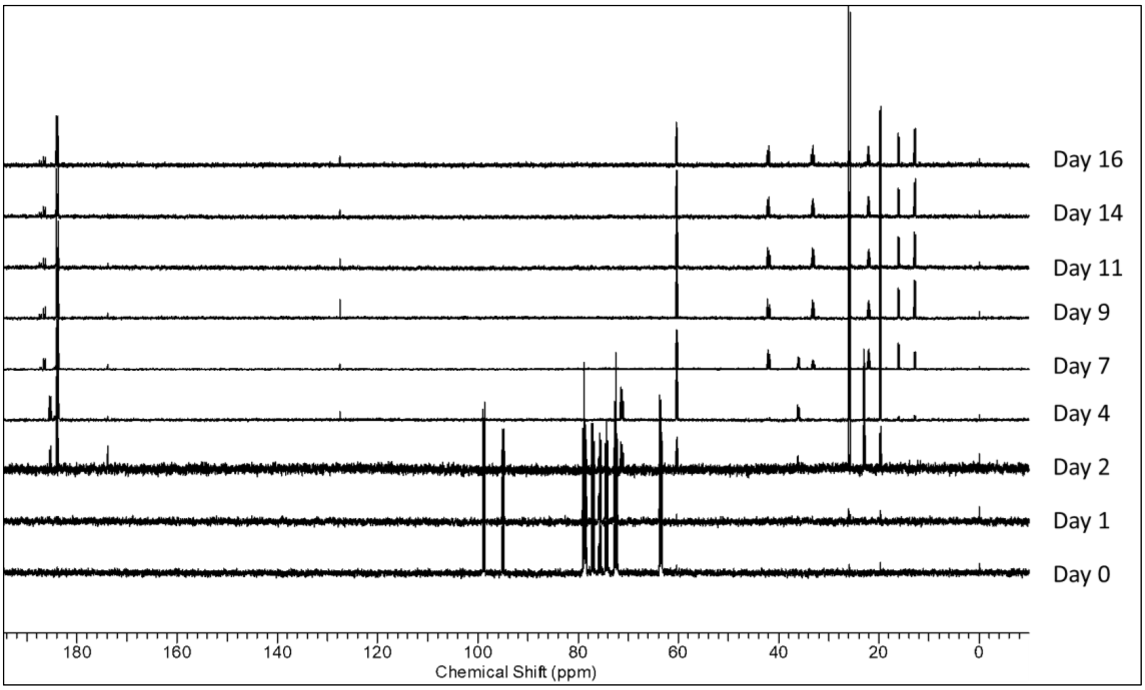

Figure S1. Superimposition of ^{13}C spectra from day 0 to day 16 of OCP MMC medium.

Table S1. Resonance assignment of ^{13}C labelled molecules in MMC medium. d: doublet; dd: doublet of doublets; s: singlet.

**Molecule**	**Assignment**	**Chemical Shift ^{13}C**	**Multiplicity**	**Coupling Constants J (Hz)**
Propionate				
[2,3-$^{13}C_2$]Propionate	β-CH_3<br>**α-CH_2**<br>C=O	12.8<br>**33.1**<br>187.5	d<br>**d**<br>d	33.24<br>52.05<br>52.05
[1,2,3-$^{13}C_3$]Propionate	β-CH_3<br>**α-CH_2**<br>C=O	12.8<br>**33.1**<br>187.5	d<br>**dd**<br>d	33.24<br>52.05<br>52.05
Propionate	**β-CH_3**<br>α-CH_2<br>C=O	**12.8**<br>33.1<br>187.5	**s**<br>s<br>s	-<br>-<br>-

Butyrate				
[1,2-$^{13}C_2$]Butyrate	C=O<br>**α-CH_2**<br>β-CH_2<br>γ-CH_3	187.5<br>**42.0**<br>21.5<br>16.1	d<br>**d**<br>d<br>s	52.02<br>52.02<br>34.68<br>34.68
[3,4-$^{13}C_2$]Butyrate	C=O<br>α-CH_2<br>**β-CH_2**<br>γ-CH_3	187.5<br>42.0<br>**21.5**<br>16.1	s<br>d<br>**d**<br>d	52.02<br>52.02<br>34.68<br>34.68
[1,2,3,4-$^{13}C_4$]Butyrate	C=O<br>α-CH_2<br>**β-CH_2**<br>γ-CH_3	187.5<br>42.0<br>**21.5**<br>16.1	d<br>dd<br>**dd**<br>d	52.02<br>52.02<br>34.68; 52.02<br>34.68
Butyrate	C=O<br>α-CH_2<br>β-CH_2<br>**γ-CH_3**	187.5<br>42.0<br>21.5<br>**16.1**	s<br>s<br>s<br>**s**	-<br>-<br>-<br>-
Acetate				
[1,2-$^{13}C_2$]Acetate	C=O<br>**α-CH_3**	183.9<br>**25.9**	d<br>**d**	53.47<br>52.02
Acetate	C=O<br>**α-CH_3**	183.9<br>**25.9**	s<br>**s**	-<br>-
Lactate				
[1,2,3-$^{13}C_3$]Lactate	C=O<br>**α-CH**<br>β-CH_3	185.3<br>**71.3**<br>23.0	d<br>**dd**<br>d	54.91<br>37.57; 54.91<br>37.57
[2,3-$^{13}C_3$]Lactate	C=O<br>**α-CH**<br>β-CH_3	185.3<br>**71.3**<br>23.0	d<br>**d**<br>d	54.91<br>37.57<br>37.57
Lactate	C=O<br>α-CH<br>**β-CH_3**	185.3<br>71.3<br>**23.0**	s<br>s<br>**s**	-<br>-<br>-
Succinate				
[1,2,3-$^{13}C_3$]Succinate	C=O<br>α-CH_2<br>**β-CH_2**<br>C=O	174.4<br>36.1<br>**36.1**<br>174.4	d<br>dd<br>**d**<br>s	50.58<br>33.24; 50.58<br>33.24<br>-

Succinate*	C=O<br>α-CH_2<br>β-CH_2<br>C=O	174.4<br>36.1<br>36.1<br>174.4	s<br>s<br>s<br>s	-<br>-<br>-<br>-
Ethanol				
[1,2-$^{13}C_2$]Ethanol	α-CH_2<br>**β-CH_3**	60.3<br>**19.7**	d<br>**d**	36.13<br>36.13
Ethanol	α-CH_2<br>**β-CH_3**	60.3<br>**19.7**	s<br>**s**	-<br>-
Glucose				
[1,2,3,4,5,6-$^{13}C_6$]α-D-Glucose	1-CH<br>2-CH<br>3-CH<br>4-CH<br>5-CH<br>6-CH	94.93<br>74.19<br>75.63<br>72.34<br>74.13<br>**63.41**	d<br>d<br>d<br>d<br>d<br>d	
[1,2,3,4,5,6-$^{13}C_6$]β-D-Glucose	1-CH<br>2-CH<br>3-CH<br>4-CH<br>5-CH<br>6-CH	98.71<br>76.95<br>78.57<br>72.34<br>72.34<br>**63.47**	d<br>d<br>d<br>d<br>d<br>d	

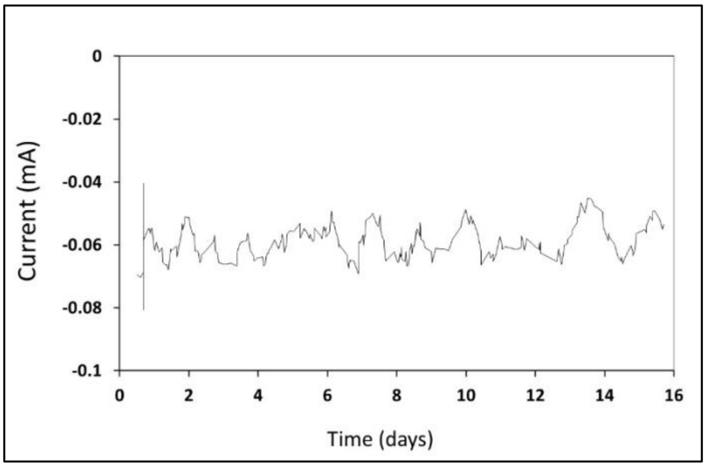

Figure S2. Characteristic current vs time profile recorded during a CEF experiment

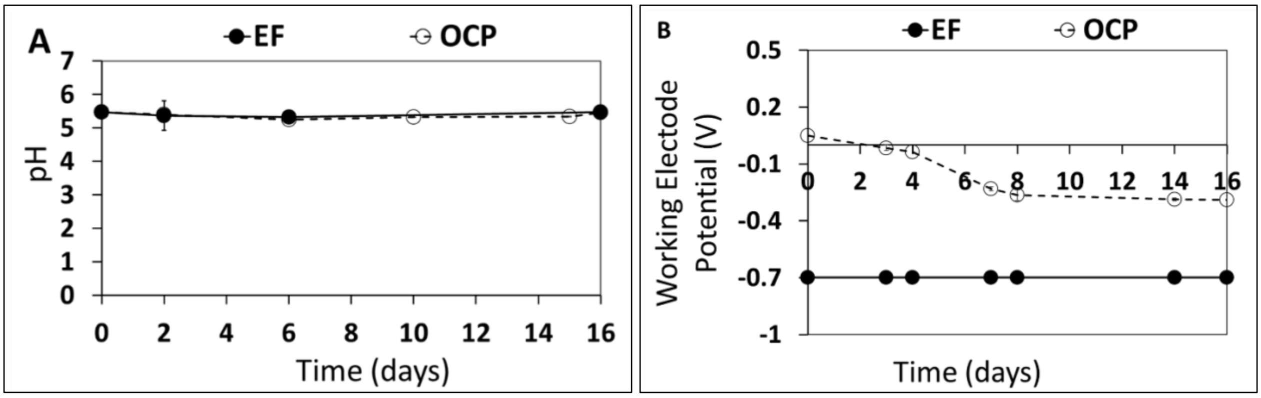

Figure S3. Time course of the pH (A) in the EF and OCP tests and the profiles of the working electrode potential during the electro-fermentation and OCP tests. Error bars represent standard deviations of duplicate experiments.

Carbon and Electron balances

Carbon balance

^{13}C glucose (mmol-C) = labelled fermented products (mmol-C) + X (mmol-C) + CO_2 (mmol-C)

Electron Balances:

^{13}C glucose (mmol-C)* DoR + *I* (mmol e^-)= labelled fermented products (mmol-C)*DoR + X (mmol-C)*DoR

where:

Labelled fermented product = labelled ethanol + labelled acetate + labelled propionate + labelled butyrate

X = Biomass growth (i.e., $C_5H_7NO_2$)

DoR= Degree of Reduction

I = mmol e- deriving from the cathodic current flowing in the system, calculated as the ratio between the overall charge (in units of Coulomb) measured during the EF tests and the Faraday constant (F= 96485 Coulomb/mol e^-). This term of the balance is obviously null for the OCP tests

The DoR for each compound was determined as follows:

DoR Glucose = 24 (mmol e^-)/6 (mmol-C) =4 (mmol e^-)/(mmol-C)

DoR Ethanol=12 (mmol e^-)/2(mmol-C) =6 (mmol e^-)/(mmol-C)

DoR Acetate=8 (mmol e^-)/2 (mmol-C) =4 (mmol e^-)/(mmol-C)

DoR Propionate =14 (mmol e^-)/3 (mmol-C) =4.67 (mmol e^-)/(mmol-C)

DoR Butyrate =20 (mmol e^-)/4 (mmol-C)=5 (mmol e^-)/(mmol-C)

*DoR X = 20(mmol e^-)/5 (mmol-C)=4 (mmol e^-)/(mmol-C)

*For the determination of the biomass Degree of Reduction (DoR X), the formula “$C_5H_7NO_2$” was considered and the calculation was done as reported previously (*Kracke and Krömer, **2014**. Identifying target processes for microbial electrosynthesis by elementary mode analysis. BMC Bioinformatics 15: 1–14.* https://doi.org/10.1186/s12859-014-0410-2).

Both carbon and electron balances were calculated using data collected on day 14th of operation, in correspondence to the second maximum observed for the fermented products. The balances are represented in **Figure S4** below, which refers to average data collected from duplicate experiments.

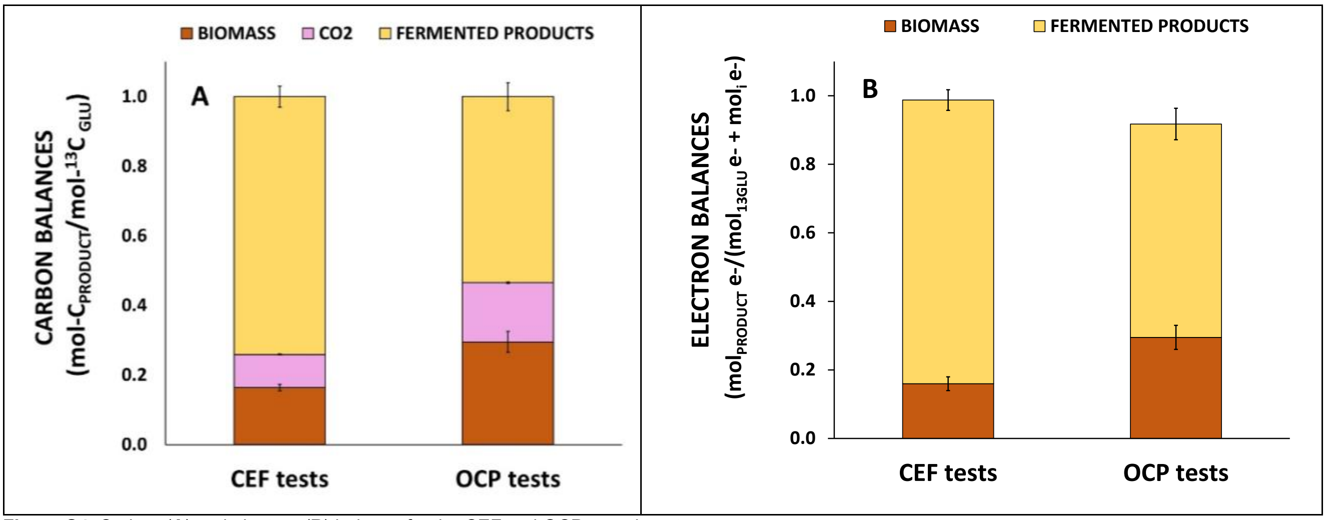

Figure S4. Carbon (**A**) and electron (**B**) balance for the CEF and OCP experiments.
